# Infection prevention and antibiotic stewardship program needs and practices in 2021: A survey of the Society for Healthcare Epidemiology of America Research Network

**DOI:** 10.1017/ice.2022.222

**Published:** 2023-06

**Authors:** KC Coffey, Sara C. Keller, Deverick J. Anderson, Valerie M. Deloney, Anthony D. Harris, David Henderson, Aaron M. Milstone, Daniel J. Morgan, Clare Rock, Marin Schweizer, Kathleen Chiotos

**Affiliations:** 1 Department of Epidemiology and Public Health, University of Maryland School of Medicine, Baltimore, Maryland; 2 Division of Infectious Diseases, Department of Medicine, Johns Hopkins University School of Medicine, Baltimore, Maryland; 3 Division of Infectious Diseases, Department of Medicine, Duke University School of Medicine, Durham, North Carolina; 4 The Society for Healthcare Epidemiology of America, Arlington, Virginia; 5 Clinical Center, National Institutes of Health, Bethesda, Maryland; 6 Division of Infectious Diseases, Department of Pediatrics, The Johns Hopkins University School of Medicine, Baltimore, Maryland; 7 Department of Internal Medicine, University of Wisconsin, Madison, Wisconsin; 8 Division of Critical Care Medicine, Department of Anesthesiology and Critical Care Medicine, Children’s Hospital of Philadelphia, Philadelphia, Pennsylvania

## Abstract

In total, 50 healthcare facilities completed a survey in 2021 to characterize changes in infection prevention and control and antibiotic stewardship practices. Notable findings include sustained surveillance for multidrug-resistant organisms but decreased use of human resource-intensive interventions compared to previous surveys in 2013 and 2018 conducted prior to the COVID-19 pandemic.

Healthcare-associated infections (HAIs) and antibiotic-resistant organisms pose significant risk to patients. In 2015, an estimated 687,000 HAIs occurred in US acute-care hospitals,^1^ and each year, >2.8 million antibiotic-resistant infections occur in the United States.^2^ To improve patient safety, the Centers for Medicare and Medicaid Services (CMS) require surveillance and reporting of HAIs, and hospitals may be rewarded or penalized in response to their HAI metrics. Furthermore, The Joint Commission implemented an antimicrobial stewardship standard for hospital accreditation programs in 2017, and in 2020, implementation of an antimicrobial stewardship program (ASP) became a CMS condition of participation.^3^ However, hospitals may vary in data collected and publicly reported, as well as in stewardship strategies used.

The Society for Healthcare Epidemiology of America Research Network (SRN) periodically performs surveys evaluating key infection prevention and control (IPC) and ASP practices among SRN-affiliated facilities.^4,5^ This report of the 2021 survey (Supplementary Fig. 1 online) updates findings from 2013 and 2018.

## Methods

The SRN is a consortium of 91 healthcare facilities from around the world that collaborate on research studies^6^ related to healthcare epidemiology and antimicrobial stewardship.^7^ SRN facilities were invited to participate in this survey between October 6, 2021, and November 26, 2021. The 15-item survey was completed by 1 principal investigator at each site using the online survey platform Alchemer (Louisville, CO). All duplicate responses were excluded; unanswered questions were classified as not performing the practice. Survey responses were primarily categorical, including ranked responses and Likert-scale responses. Descriptive statistical analysis was performed using Microsoft Excel software (Microsoft, Redmond, WA). This survey was not considered human subjects research.

## Results

Of the 91 facilities invited to participate, 50 (55%) completed the 2021 survey compared to 64 (48%) of 132 in 2018 and 69 (34%) of 202 in 2013. Among the respondents to the 2021 survey, 25 (50%) also completed the 2018 survey, and 16 (32%) completed all 3 surveys, in 2013, 2018, and 2021. Participating facilities were from 21 US states and at least 1 each from Canada, South Korea, India, Spain, Mexico, the United Arab Emirates, and Egypt.

### Practices

Surveillance for multidrug-resistant organisms was performed by most facilities during all 3 survey periods. Surveillance for methicillin-resistant *Staphylococcus aureus* remained most common (43 of 50, 86%). Surveillance for antibiotic-resistant gram-negative organisms, including carbapenem-resistant Enterobacterales, increased across the 3 surveys (Table 1). Surveillance for *Candida auris* was reported by 26 (52%) of 50 respondents in 2021; surveillance practices for this emerging pathogen were not measured in prior surveys.

**Table 1.** Healthcare-Associated Infection and Multidrug-Resistant Organism Surveillance and Infection Control Practices Among the 2013, 2018, and 2021 Society for Healthcare Epidemiology of America Research Network Surveys

Variable	Proportion of 2013 Respondents, No./Total Responses (%)	Proportion of 2018 Respondents (N = 64), No. (%)	Proportion of 2021 Respondents (N = 50), No. (%)
**MDRO surveillance**			
MRSA	55/61 (90)	44 (69)	43 (86)
VRE	33/61 (59)	29 (45)	38 (76)
*C. auris*	NR	NR	26 (52)
CRE	NR	32 (50)	39 (78)
GN-MDRO	28/61 (46)	32 (50)	36 (72)
**HAI surveillance**			
CLABSI	65/68 (96)	64 (100)	48 (96)
Publicly reported	NR	50 (78)	40 (80)
CAUTI	63/68 (93)	61 (95)	45 (90)
Publicly reported	NR	48 (75)	37 (74)
HO-CDI	NR	57 (89)	43 (86)
Publicly reported	NR	47 (73)	34 (68)
HOB	NR	53 (83)	42 (84)
Publicly reported	NR	44 (69)	32 (64)
SSI	64/68 (95)	62 (97)	47 (94)
Publicly reported	NR	49 (77)	35 (70)
VAE/VAP	50/68 (74)	52 (81)	37 (74)
Publicly Reported	NR	17 (27)	17 (34)
**Environmental cleaning**			
Monitoring			
Visual	31/69 (45)	44 (68)	34 (68)
UV markers	25/69 (36)	31 (48)	27 (54)
ATP detection	19/69 (28)	30 (47)	19 (38)
Other	NR	5 (8)	7 (14)
Disinfection			
UV disinfection	13/68 (19)	24 (37)	32 (64)
H2O2 vapor	6/68 (9)	16 (25)	9 (18)

Note. MDRO, multidrug-resistant organism; MRSA, methicillin-resistant *Staphylococcus aureus*; VRE, vancomycin-resistant *Enterococcus*; *C. auris*, *Candida auris*; NR, not reported; CRE, carbapenem-resistant Enterobacterales; GN-MDRO, gram-negative multidrug-resistant organism (eg, *Acinetobacter*, extended-spectrum ß-lactamase); HAI, healthcare-associated infection; CLABSI, central-line–associated bloodstream infection; CAUTI, catheter-associated urinary tract infection; HO-CDI, hospital-onset *Clostridioides difficile* infection; HOB, hospital-onset MRSA bacteremia; SSI, surgical site infection; VAE/VAP, ventilator-associated event/ventilator-associated pneumonia; CHG, chlorhexidine gluconate; UV, ultraviolet; ATP, adenosine triphosphate; H2O2, hydrogen peroxide.

Relative to the 2013 and 2018 surveys, the proportion of facilities performing surveillance for HAIs decreased slightly, and trends in public reporting of HAIs were variable. In 2021, central-line–associated bloodstream infection (CLABSI) surveillance was performed in 48 (96%) of 50 facilities, compared to 100% in 2018. In 2021, catheter-associated urinary tract infections (CAUTI) surveillance was performed in 45 (90%) of 50 facilities, compared to 61 (95%) of 64 in 2018. Similarly, in 2021, hospital-onset *Clostridioides difficile* infection (HO-CDI) surveillance was performed in 43 (86%) of 50 facilities versus 57 (89%) of 64 facilities in 2018. Also, ventilator-associated event (VAE) and/or ventilator-associated pneumonia (VAP) surveillance was performed in 37 (74%) of 50 facilities in 2021 versus 52 (81%) of 64 facilities in 2018. Of these HAIs, CLABSI and CAUTI were publicly reported by a similar proportion of facilities in 2021 (80% vs 78%) and 2018 (74% vs 75%). Public reporting of HO-CDI and SSI decreased slightly in 2021 (68% vs 73%) compared with 2018 (70% vs 77%), whereas public reporting of VAE/VAP increased slightly in 2021 (34%) compared with 2018 (27%) (Table 1). All forms of environmental cleaning surveillance increased from 2013 to 2021, but when comparing 2018 to 2021, there was an increase in the use of ultraviolet (UV) markers and a decrease in the use of adenosine triphosphate detection. UV disinfection also increased over time, whereas hydrogen peroxide disinfection use peaked in 2018 (Table 1).

Preauthorization or formulary restriction as a stewardship strategy was used by a greater proportion of facilities in 2021 (43 of 46, 93%) compared to 2018 (48 of 60, 80%). However, prospective audit with feedback was utilized less frequently in 2021 than 2018 (74% vs 93%). Staff education, pharmacy-driven interventions, and monitoring antimicrobial days of therapy were also less frequent in 2021, whereas the use of rapid diagnostic tests for bloodstream infections increased (Table 2 and Supplementary Table 1 online).

**Table 2.** ASP Activities Among Facilities Participating in One or More Core ASP Activities

ASP Strategies	2018 Respondents Participating in ASP Practices (Total n = 60), No. (%)	2021 Respondents Participating in ASP Practices (Total n = 46), No. (%)
Preauthorization/formulary restriction	48 (80)	43 (93)
Prospective audit with feedback	56 (93)	34 (74)
Monitoring antimicrobial DOT	52 (87)	35 (76)
Monitoring DDD	No data	19 (41)
Maintaining an institutional antibiogram	52 (87)	42 (91)
Maintaining at least 1 unit-specific antibiogram	No data	18 (39)
Interpretation and interventions based on rapid molecular diagnostic tests	No data	29 (63)
Providing regular education to staff	47 (78)	31 (67)
Pharmacy-driven interventions (eg, IV to PO conversion)	51 (85)	37 (80)

Note. ASP, antimicrobial stewardship program; DOT, days of therapy; DDD, defined daily doses; IV, intravenous, PO, oral.

### Challenges

The 3 greatest challenges facing IPC programs were lack of trained personnel or staffing constraints (22 of 50, 44%), insufficient time devoted to frontline staff (21 of 50, 42%), and insufficient information technology and/or data support (20 of 50, 40%). Generally, infection preventionist and hospital epidemiologist full-time equivalents (FTE) for IPC activities increased with bed size, but FTE support was variable across hospitals of all sizes. For ASPs, insufficient information technology and/or data analyst support was the greatest challenge (20 of 50, 40%), followed by insufficient financial support and insufficient time to perform work responsibilities (each 18 of 50, 36%). Regardless of hospital size, most respondents (34 of 50, 68%) reported ≤1 FTE for physician dedicated ASP activities, and only 14 (28%) of 50 had any dedicated FTE for an ASP data analyst (Supplementary Fig. 2 online). Most respondents expected overall IPC and ASP responsibilities to increase in the following year (73% and 53%, respectively), yet the majority (77% and 79%, respectively) expected no concomitant increase in FTE support (Supplementary Table 2 online).

## Discussion

This survey characterizes the IPC and ASP practices of healthcare facilities participating in the SRN. These findings update the results of the surveys conducted in 2013 and 2018. Leading challenges identified by IPC programs and ASPs in this survey were insufficient time, trained personnel, and financial support to perform work responsibilities, all of which were exacerbated by the coronavirus disease 2019 (COVID-19) pandemic.^8^ Compared to prior surveys, interventions requiring human capital, time, and specialized training (eg, prospective audit with feedback) were employed less frequently, whereas interventions utilizing technology were largely unchanged. Although no survey questions specifically asked about the impact of the COVID-19 pandemic, the excess workload and decreased human resources for routine IPC or ASP activities likely resulted from pandemic-related demands. Fleisher et al^9^ recently highlighted the pandemic as an opportunity to build a more resilient system by investing not only in person-power, training, and education but also technology, data support, and research to embed more automated solutions that may be relied on during staffing crises. The results of our survey reinforce this opportunity and suggest that a shift toward less time-intensive techniques may help programs maintain patient safety standards across a wider breadth of activities.

This study had several limitations. First, comparisons to the 2013 and 2018 surveys may be imperfect because the same institutions have not always responded. However, even if the respondents are different, the SRN represents geographically diverse institutions reflecting the broader healthcare-epidemiology community. Second, to adapt to changing priorities in healthcare epidemiology, the survey questions have been revised slightly over time, precluding comparisons for all items.

Overall, the 2021 SRN survey results have emphasized the challenges facing IPC programs and ASPs, underscoring the lack of trained personnel and time to complete key activities. Although personnel and funding have been among the 3 greatest challenges identified in IPC and ASP throughout periodic surveys, the 2021 survey results suggest a need for infrastructure that offloads time-intensive practices when staff resources are at a premium. Regulatory bodies may consider revising HAI and stewardship metrics to capture meaningful data that rely less on personnel time, and hospitals should invest in automated data collection, analysis, and reporting to liberate person-power to frontline activities. Finally, government agency funding at all levels is needed to support recruitment, training, and ongoing research to guide best practices and create a more resilient system.

## Supplementary material

For supplementary material accompanying this paper visit https://doi.org/10.1017/ice.2022.222. click here to view supplementary material
