# Supplementary material for: Infection prevention and antibiotic stewardship program needs and practices in 2021: A survey of the Society for Healthcare Epidemiology of America Research Network
Source: Infect Control Hosp Epidemiol. 2023 Mar 14;44(6):948–50. doi: 10.1017/ice.2022.222 (PMC10262154; doi:10.1017/ice.2022.222)
Supplement: Supplementary file 1 [file S0899823X22002227sup001.docx]

**Supplemental Figure 1. State of the SRN in 2021: Infection Prevention and Antibiotic Stewardship Programs and Practices**

## State of the SRN in 2021: Infection Prevention and Antibiotic Stewardship Programs and Practices

#### What are the top 3 challenges for your organization’s infection prevention and control (IPC) program?

- Insufficient financial support (please briefly describe):
- Insufficient clinical microbiology support
- Insufficient information technology/data support
- Insufficient non-financial support by leadership or lack of leadership engagement (please briefly describe):
- Lack of research to support IPC strategies
- Lack of trained personnel or staffing constraints independent of financial support
- Lack of support for continuing education
- Insufficient time for staff to perform work responsibilities
- Insufficient time devoted to frontline staff (i.e. education, rounds)
- No challenges
- Other - Write In:

#### Which healthcare-associated infections (HAIs) or multidrug-resistant organisms (MDROs) does your facility perform surveillance and/or publicly report? Check all that apply and enter any not listed in the text box below.

- Central line-associated bloodstream infection (CLABSI)
- Catheter-associated urinary tract infection (CAUTI)
- *C. auris*
- *C. difficile* infection (CDI)
- Hospital-onset CDI
- Carbapenem-resistant Enterobacterales
- Gram-negative MDROs (e.g. Acinetobacter, ESBL)
- Methicillin-resistant *S. aureus* (MRSA)

1. **How much hospital financial support do hospital epidemiologists and infection preventionists receive? If there is more than one individual, please select the sum of all dedicated FTE.**

|  | (0-0.5 FTE, 0.6-1 FTE, 1.1-1.5 FTE, 1.6-2 FTE, 2.1-2.5 FTE, 2.6-3 FTE, 3.1-3.5 FTE, 3.6-4 FTE. 4.1-4.5 FTE, 4.6-5 FTE, 5.1-5.5 FTE, 5.6-6 FTE, 6.1-6.5 FTE, 6.6-7 FTE, 7.1-7.5 FTE, 7.6-8 FTE, 8.1-8.5 FTE, 8.6-9 FTE, 9.1-9.5 FTE, 9.6-10 FT, >10 FTE, don't know) |
| --- | --- |
| Hospital epidemiologist  Infection preventionist |  |

#### Does your facility have a chlorhexidine gluconate (CHG) bathing policy in at least one unit?

- Yes
- No
- Don't know

#### Which methods or tools does your facility use for environmental monitoring? Check all that apply or "none."

- Visual inspection checklist
- UV visible fluorescent markers
- ATPase devices
- None
- Other - Write In:

#### Which technologies does your facility use for environmental cleaning and disinfection? Check all that apply or "none."

- UV disinfection device
- Hydrogen peroxide vapor
- None
- Other - Write In

#### Does your facility have policies that require HCP to be vaccinated against seasonal flu and/or COVID-19?

|  | (Yes, all HCP must be vaccinated with exemptions only for medical contraindications or other federal/state requirements (e.g. religious); Yes, with exemptions in addition to medical contraindications or other federal/state requirements; Vaccination is not a condition of employment, but declination forms are required; No, there is no requirement for HCP to receive this vaccine) |
| --- | --- |
| Seasonal fluCOVID-19 |  |

### If you have a comment or clarification about your facility's HCP vaccination policies, enter it here:

### What are the top 3 challenges for your organization’s antimicrobial stewardship program (ASP)?

- Insufficient financial support (please briefly describe):
- Insufficient clinical microbiology support
- Insufficient time devoted to frontline staff (i.e. education, rounds)
- Insufficient information technology/data support
- Insufficient non-financial support by leadership or lack of leadership engagement (please briefly describe):
- Lack of research to support antimicrobial stewardship strategies
- Lack of trained personnel/staffing constraints, independent of financial support
- Lack of support for continuing education
- Insufficient time for staff to perform work responsibilities
- Insufficient time devoted to frontline staff (i.e. education, rounds)
- No challenges
- Other - Write In:

1. **How much hospital financial support do these personnel receive for ASP activities? If there is more than one individual, please select the sum of all dedicated FTE.**

|  | (No financial support from hospital for ASP activities; 0-0.5 FTE; 0.6-1 FTE; 1.1-1.5 FTE; 1.6-2 FTE; 2.1-2.5 FTE; 2.6-3 FTE; 3.1-3.5 FTE; 3.6-4 FTE; 4.1-4.5 FTE; 4.6-5 FTE; >5 FTE; Don't know) |
| --- | --- |
| Physician  Nurse practitioner or physician assistant  ID residency trained clinical pharmacist  Non-ID residency trained clinical pharmacist  Nurse  Data analyst |  |

#### Does the ASP provide antimicrobial prescribing guidelines or pathways for at least one common infectious condition?

- Yes
- No
- Don't know
- Not applicable

#### Which of the following conditions receives antimicrobial recommendations by the ASP? Check all that apply.

- Abdominal infections
- Bacteremia
- Candidiasis in the non-neutropenic patient
- Central nervous system (CNS) infections
- Endovascular infections and endovascular device infections
- Gastrointestinal infections
- Gynecologic and sexually transmitted infections
- Head and neck infections
- Malaria
- Febrile neutropenia
- Pulmonary infections
- Sepsis with no clear source
- Skin, soft-tissue, and bone infections
- Urinary tract infections
- Not applicable
- Other - Write In:

#### Which of the following strategies are utilized by your ASP? Check all that apply.

- Preauthorization/formulary restriction
- Prospective audit with feedback
- Monitoring antimicrobial days of therapy (DOT)
- Monitoring defined daily doses (DDD)
- Maintaining an institutional antibiogram
- Maintaining at least one unit-specific antibiogram
- Interpretation of and interventions based on rapid molecular diagnostic tests
- Providing regular education to staff
- Pharmacy-driven interventions (for example, IV to PO conversion, dose optimization)
- Not applicable
- Other - Write In:

#### Does your facility use rapid diagnostic tests on blood culture specimens as part of antimicrobial stewardship activities? Check all that apply.

- MALDI-TOF MS
- *S. aureus* PCR (Xpert MRSA/SA, BD Max StaphSR Assay)
- Staph PNA FISH/QuickFISH
- Enterococcus PNA FISH/QuickFISH
- Verigene BC-GP
- Verigene BC-GN
- GenMark ePlex BCID Gram-Negative (BCID-GN) Panel
- GeneMark ePlex BCID Fungal-Pathogen (BCID-FP) Panel
- Gram-negative PNA FISH/Quick FISH
- Film Array Blood Culture Identification Panel (BCID, BioFire)
- Accelerate PhenoTest BC
- T2 *C. auris* panel
- Not applicable
- Other - Write In:

#### How do you expect the following to change next year:

|  | (Increasing; Staying the same; Decreasing; Not applicable) |
| --- | --- |
| Overall IPC responsibilitiesOverall ASP responsibilitiesReporting requirementsResearch in infection preventionResearch in antimicrobial stewardshipMeetingsTime devoted to frontline staff (rounds, education)IPC FTEs (including medical director, IP, analyst)ASP FTEs (including medical director, clinical pharmacist, analyst) |  |

**Supplemental Table 1. Rapid Molecular Diagnostic Tests Used for Diagnosis of Bacteremia Among Respondents Utilizing One or More Rapid Diagnostic Test**

| **Rapid diagnostic tests for bacteremia** | **2018 Respondents using Rapid Diagnostics, No (%)**  **(Total n=60)** | **2021 Respondents using Rapid Diagnostics, No. (%)**  **(Total n=44)** |
| --- | --- | --- |
| MALDI-TOF | 30 (50) | 36 (82) |
| *Staphylococcus aureus* PCR | 16 (27) | 25 (57) |
| *S. aureus* PNA FISH/QuickFISH | 1 (2) | 0 (0) |
| *Enterococcus* PNA FISH/QuickFISH | 1 (2) | 2 (5) |
| Verigene BC gram positive | 15 (25) | 12 (27) |
| Verigene BC gram negative | 12 (20) | 11 (25) |
| GenMark ePlex BCID Gram-Negative | No data | 6 (14) |
| GenMark ePlex BCID Fungal-Pathogen | No data | 4 (9) |
| Gram-negative PNA FISH/Quick FISH | 3 (5) | 0 (0) |
| Film Array Blood Culture Identification Panel | 17 (28) | 18 (41) |
| Accelerate PhenoTest BC | 1 (2) | 2 (5) |
| T2 *Candida* panel | 3 (5) | 1 (2)^1^ |

^1^*C. auris* panel

**Supplemental Figure 2. Full-time Equivalents (FTE) Dedicated to IPC and ASP by Hospital Size**

**Supplemental Table 2. Projected Infection Prevention and Control and Antibiotic Stewardship Responsibilities and Resources**

|  | **Increase**  **% (No./Total Responses)** | **Decrease**  **% (No./Total Responses)** | **Stay the Same**  **% (No./Total Responses)** |
| --- | --- | --- | --- |
| Reporting requirements | 54 (26/48) | 0 (0/48) | 44 (21/48) |
| Meetings | 40 (19/48) | 6 (3/48) | 54 (26/48) |
| Time devoted to frontline staff (rounds, education) | 33 (16/48) | 10 (5/48) | 54 (26/48) |
| *Infection Prevention and Control (IPC)* | | | |
| Overall Responsibilities | 73 (35/48) | 0 (0/48) | 27 (13/48) |
| Research in infection prevention | 38 (18/48) | 15 (7/48) | 44 (21/48) |
| Full-time equivalents (FTE) including medical director, infection preventionist, analyst | 23 (11/48) | 0 (0/48) | 77 (37/48) |
| *Antibiotic Stewardship Program (ASP)* | | | |
| Overall Responsibilities | 53 (25/47) | 0 (0/47) | 43 (20/47) |
| Research in antimicrobial stewardship | 34 (16/47) | 6 (3/47) | 47 (22/47) |
| Full-time equivalents (FTE) including medical director, clinical pharmacist, analyst | 13 (6/47) | 4 (2/47) | 79 (37/47) |
